# Reduction of missed appointments at an urban primary care clinic: a randomised controlled study

**DOI:** 10.1186/1471-2296-11-79

**Published:** 2010-10-25

**Authors:** Noelle Junod Perron, Melissa Dominicé Dao, Michel P Kossovsky, Valerie Miserez, Carmen Chuard, Alexandra Calmy, Jean-Michel Gaspoz

**Affiliations:** 1 Division of primary care, Department of community medicine and primary care, Geneva University Hospitals, 4 rue Gabrielle Perret-Gentil, 1211 Geneva 14, Switzerland; 2 HIV Unit, Department of internal medicine, Geneva University Hospitals, 4 rue Gabrielle Perret-Gentil, 1211 Geneva 14, Switzerland

## Abstract

**Background:**

Missed appointments are known to interfere with appropriate care and to misspend medical and administrative resources. The aim of this study was to test the effectiveness of a sequential intervention reminding patients of their upcoming appointment and to identify the profile of patients missing their appointments.

**Methods:**

We conducted a randomised controlled study in an urban primary care clinic at the Geneva University Hospitals serving a majority of vulnerable patients. All patients booked in a primary care or HIV clinic at the Geneva University Hospitals were sent a reminder 48 hrs prior to their appointment according to the following sequential intervention: 1. Phone call (fixed or mobile) reminder; 2. If no phone response: a Short Message Service (SMS) reminder; 3. If no available mobile phone number: a postal reminder. The rate of missed appointment, the cost of the intervention, and the profile of patients missing their appointment were recorded.

**Results:**

2123 patients were included: 1052 in the intervention group, 1071 in the control group. Only 61.7% patients had a mobile phone recorded at the clinic. The sequential intervention significantly reduced the rate of missed appointments: 11.4% (n = 122) in the control group and 7.8% (n = 82) in the intervention group (p < 0.005), and allowed to reallocate 28% of cancelled appointments. It also proved to be cost effective in providing a total net benefit of 1846. - EUR/3 months. A satisfaction survey conducted with 241 patients showed that 93% of them were not bothered by the reminders and 78% considered them to be useful. By multivariate analysis, the following characteristics were significant predictors of missed appointments: younger age (OR per additional decade 0.82; CI 0.71-0.94), male gender (OR 1.72; CI 1.18-2.50), follow-up appointment >1year (OR 2.2; CI: 1.15-4.2), substance abuse (2.09, CI 1.21-3.61), and being an asylum seeker (OR 2.73: CI 1.22-6.09).

**Conclusion:**

A practical reminder system can significantly increase patient attendance at medical outpatient clinics. An intervention focused on specific patient characteristics could further increase the effectiveness of appointment reminders.

## Background

Missed appointments are known to interfere with appropriate care of acute and chronic health conditions and to misspend medical and administrative resources. They represent a major burden on health care systems and costs [1,2], by reducing the effectiveness of outpatient health care delivery. This leads to suboptimal use of clinical and administrative staff, increases waiting times for other patients, and affects continuity of care. Prevalence of missed appointments ranges from 5 to 55% and varies between countries, health care systems and clinical settings [3-6]. Reasons given for missing a medical appointment have been widely analysed and include factors such as forgetfulness, feeling better or worse, transport problems and misunderstanding/confusion about the time of consultation [7-10].

Several interventions have been tested to reduce the rate of missed appointments. Telephone reminders are effective and give patients the opportunity to cancel or to postpone their appointment [11-13]. Postal reminders are effective to a lesser extent, and their effect tends to decrease with time [9]. Use of short message service (SMS) improves attendance [14]. More recently, randomised controlled studies comparing SMS, phone calls and no intervention showed that SMS and phone reminders were equally effective in reducing the rate of missed appointments, SMS being more cost-effective [15,16]. However, patients are often reluctant to give their cell phone number to medical practices and the cell phone penetration in a given population may be lower than expected.

In the primary care clinic of the Geneva University Hospitals, the rate of missed appointments was evaluated at 22% in 2007. Strategies implemented a year before the study, such as charging 20 EUR for each missed appointment and overbooking, led to no changes. The aim of the study was to test the effectiveness of a reminder on the rate of missed appointments in our clinic, and to determine the profile of non attenders. Given the fact that most of our patients belong to vulnerable population groups and often have incomplete contact information, we decided to conduct a sequential reminder intervention using successive recommended strategies. We expected a higher rate of non attendance among undocumented immigrants and for new consultations or post-emergency department visits.

## Methods

### Setting

The study was conducted at the primary care clinic and ambulatory HIV clinic of the Geneva University Hospitals, Switzerland. The primary care clinic provides ambulatory care to approximately 12 000 scheduled consultations per year for patients over the 16 years of age. Care is delivered mostly by junior doctors (n = 18-20), enrolled in a 12 to 24-month training program in general practice after two-three years of inpatient care, at the end of their training in primary care. Consultations include general consultations, tobacco cessation and dietician consultations, provided by trained physicians and a nutritionist, respectively. The HIV clinic offers follow-up consultations to about 600 patients per year and is run by 2.4 full time-equivalent junior doctors and one full time senior physician. These two clinics are known to care for vulnerable patient populations including undocumented migrants, asylum seekers, patients without proper insurance coverage and legal immigrants. These patients often have changing or incomplete contact information and are reluctant to disclose such information, rendering them difficult to reach. A previous study showed that half of the patients attending the primary care clinic were immigrants and that 40% of patients did not speak French [17].

### Patients

All patients scheduled to attend these two clinics between April and June 2008 were included if their consultation had been booked more than 48 hours before the due date. In our setting, patients are usually requested to give their phone number in case appointments need to be changed. Their phone number is written on the electronic appointment record of the clinic. Such a request was systematically made to all patients during the study period. Patients were informed of the study by signposting in the waiting rooms and at the reception desk, and orally when the appointment was made.

### Randomisation

An investigator (MPK), who was not involved in patient recruitment, created a randomisation sequence using a computerised random-number generator. The research assistant (CC) daily randomised consecutive patients into two groups on the basis of a printed version of the electronic appointment record, using the computer generated sequence.

### Intervention group

Because of our population specificities, we decided to conduct a sequential intervention starting 48 hours before the appointment. The research assistant applied the following intervention: first a phone call, mobile or fixed according to the number written on the electronic appointment record; second, a SMS (text messaging) if participants did not answer the phone after 3 attempts and had a mobile phone; and finally a postal reminder if participants did not answer the phone, had no mobile phone for SMS, or had no phone at all. The postal reminder would reach the patient the next day. Such a design was selected in order to reach a maximum of patients through different reminder systems, while giving the opportunity to a majority of patients to immediately cancel or postpone an appointment, thus allowing us to reallocate consultations [11]. Languages used by the research assistant for the phone calls were French, English or Spanish. The SMS was sent in French and would include the name of the physician, the day and time of the appointment, but no medical information. No information was given over the phone if the patient was not personally reached.

### Control group

In the control group, patients did not receive any reminder about their upcoming appointment.

The study was approved by the hospital's research ethics committee.

### Outcomes

The primary outcome measure was the rate of missed appointment in the clinics (general, tobacco cessation, HIV, and dietician consultations). Appointments were defined as "missed" if patients did not come to the consultation without informing the clinic. Other measures included were: 1) the number of reported, cancelled and rebooked appointments during the intervention in both groups; 2) the percentage of patients having a fixed phone, a mobile phone or no phone; 3) the process of the intervention: mean of communication used, number of attempts, time of the day when patients were reached, number of refusals; 4) the cost-effectiveness of the intervention; and 5) the sociodemographic and medical profile of patients missing their appointments, including information about age, gender, nationality, legal status, health insurance status, type of consultation (new, post-emergency department visit (ED) or follow-up), physician status (junior or senior doctor), presence or absence of psychiatric diagnoses. We finally conducted a Likert-type evaluation of the usefulness and the acceptability of the intervention through a phone survey to a randomly selected sample of patients.

The study was conducted during three months.

### Statistical analysis

The sample size defined to observe a reduction in the rate of missed appointments from 20% to 10% was calculated with a power of 0.90 and a p < 0.05. It required including 250-300 consultations in the « intervention » group and 250-300 in the « control » group. However, half of our consultations being dedicated to undocumented immigrants presenting a different sociodemographic profile from the rest of our population (majority of South American women speaking only Spanish, with no official home), we decided to also measure the impact of the intervention in this specific sub-group of patients and, thus, to double the sample size to obtain a minimum of 600 patients in each group. The statistical analysis was made on an "intention to treat". We compared patient characteristics in both groups by means of Fisher exact tests for categorical variables and Mann Whitney tests for continuous variables. Continuous variables were expressed as mean and standard deviation (SD), categorical variables as number and percentage. P values of 0.05 or less were considered statistically significant.

In order to determine which variables were associated with a missed appointment, we built a multivariable logistic regression model in which patient characteristics were introduced. In order to assess which variables were associated or not with a missed appointment, we kept all the variables in the model, even though several of them did not reach statistical significance.

All analyses were performed with Stata release 10 (Stata Corporation, College Station, TX).

## Results

**Figure 1:**
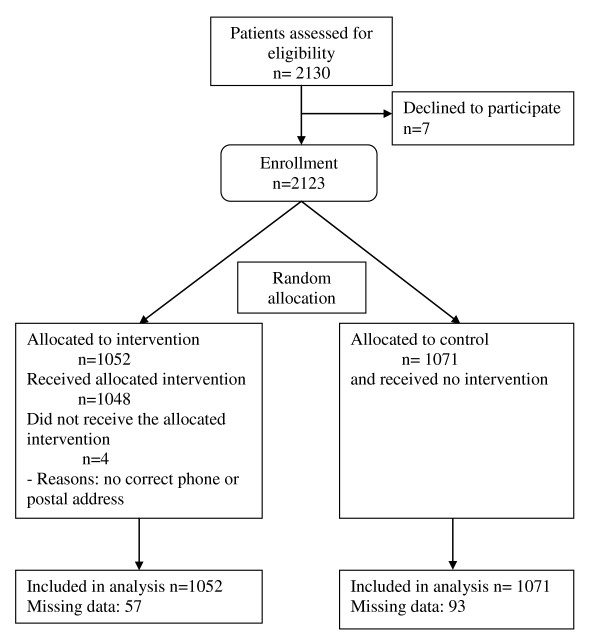
**Participant flow chart**.

2130 patients were included in the study. Seventy five percent of patients came from the general consultation (n = 1600); 6% from the smoking cessation consultation (n = 127), 4% from the dietician consultation (n = 127) and 15% from the HIV consultation (n = 303). Before randomisation, 7 patients refused to be included into the study. After randomisation, 1071 patients (50.5%) were included into the control group and 1052 (49.5%) into the intervention group. Patients' characteristics are summarised in Table 1. The randomisation process is summarised in Figure 1. Groups were well balanced; mean age was 46 and 47 years in both groups, and 22% of patients had no health insurance.

**Table 1:** Demographic and clinical characteristics of the control and intervention groups (% or standard deviation)

	Control n = 1071	Intervention n = 1052	P value
Men	607 (57%)	571 (54%)	0.31
Mean age	45.7 (SD 14.3)	46.7 (SD 22.0)	0.45
Asylum seeker	31 (2.9%)	30 (2.8%)	1.0
Uninsured	245 (22.9%)	226 (21.5%)	0.47
Type of follow-up			
New	102 (9.5%)	93 (8.8%)	
<1yr	652 (60.9%)	660 (62.7)	
>1 yr	40 (3.7%)	38 (3.6%)	
Post ED visit	31 (2.9%)	27 (2.6%)	0.92
Health care provider			
Junior doctor	626 (58.5%)	641 (60.9%)	
Senior doctor	117 (10.9%)	112 (10.7%)	
Dietician	55 (5.1%)	34 (3.2%)	0.16
Specific comorbidities:			
Depression	179 (16.7%)	155 (14.7%)	0.21
Psychosis	9 (0.8%)	15 (1.4%)	0.23
Addiction	51 (4.8%)	70 (6.7%)	0.06

ED = Emergency department

In the intervention group, 55.7% (n = 586) of patients had a mobile phone recorded at the clinic, 25.6% (n = 269) a fixed phone, 6% (n = 63) both a fixed and a mobile phone and finally 12% (n = 127) no phone. Altogether, only 61.7% (n = 586 + 63) reported having a mobile phone.

### The intervention process

1016 patients were contacted: 535 (51%) on their mobile phone, 195 (19%) on their fixed phone, 188 by SMS (18%) and 98 (10%) by post. Most patients were reached by telephone between 10 am and 12 am (n = 471). Seventy six percent of the patients with a mobile phone and 69% of patients with a fixed phone were reached after the first attempt.

Information on the effectiveness of the intervention is lacking in 93 (8.7%) patients in the control group and 57 (5.4%) in the intervention group: 50 (4.7%) in the control group and 27 (2.6%) in the intervention group were deleted from the electronic scheduling records without clear reasons (probably by doctors directly cancelling or reporting appointments without informing the receptionists), and data were not collected in 43 (4%) and 30 (2.9%) remaining patients of both groups, respectively.

In the intervention group, 54 of the 730 (7.4%) patients reminded by telephone 48 hours before cancelled their appointments during the phone call. Fifteen of these 54 (27.8%) newly available scheduling slots were reallocated to other patients. Otherwise, 26 consultations were cancelled less than 24 hours before the appointment in the intervention group and 56 in the control group. Reallocation of free slots was not recorded for these two groups, but this occurrence was considered very rare.

### Effectiveness of the intervention

Overall, the intervention significantly reduced the rate of missed appointments from 122/1071 (11.4%) to 82/1052 (7.8%; p < 0.005) (Table 2). However, subgroup analysis showed that the decrease in missed appointments was statistically significant in only two consultations: the general and the smoking cessation consultations. The decrease of missed appointment failed to reach statistical significance in the HIV clinic, as well as at the dietician consultation (p = 0.62 and 0.75, respectively).

**Table 2:** Proportion of missed appointments in the control and intervention groups

	Control group (n = 1071)	Intervention group (n = 1052)	p*
Total	122 (11.4%)	82 (7.8%)	0.005
General consults	79 (9.8%)	52 (6.5%)	0.017
HIV	23 (15%)	19 (12.7%)	0.62
Smoking cessation	15 (22.4%)	5 (8.3%)	0.049
Dietician	6 (11%)	5 (13.2%)	0.75

* Fisher exact

### Sociodemographic and medical profile of non attenders

A multivariate regression logistic analysis including all parameters known to potentially influence the rate of attendance showed that being young, male, asylum seeker having a follow-up appointment of more than one year, being cared for by a junior doctor rather than by a senior doctor, and having substance abuse problems were significantly associated with missed appointments (Table 3). There was no increased odds of missed appointments for variables such as undocumented migrants, non French speaking patients, new patients, or post ED visits. Being part of the intervention decreased the risk of missing an appointment for all categories of patients found at risk (OR 0.63, CI 0.43-0.89).

**Table 3:** Likelihood of missing an appointment according to patient characteristics, type of consultations, and health care provider status

Variables	Odds Ratio	95%CI
**Sociodemographic data**		
Age (per additional decade)	**0.82**	0.71-0.94
Male	**1.72**	1.18-2.50
Asylum seeker	**2.73**	1.22-6.09
No health insurance	1.06	0.69-1.62

**Type of consultation**		
New patient	0.50	0.22-1.12
Follow-up > 1 year	**2.2**	1.15-4.2
Post ED visit	1.54	0.66-3.55

**Identity of the health care provider**		
Junior doctor	1.00	
Senior doctor	**0.50**	0.27-0.93
Dietician	0.89	0.37-2.17

**Medical condition**		
Depression	1.48	0.97-2.25
Psychosis	0.38	0.05-2.95
Addiction	**2.09**	1.21-3.61

Being part of the intervention group	0.63	0.43-0.89

ED = Emergency Department

### Acceptability of the intervention

The satisfaction survey included 241 randomly selected patients from the intervention group, of whom 212 (78%) had received the reminder via a phone call. 224 (93%) were not disturbed by the reminder; 189 (78%) considered it to be useful; 165 (69%) recommended it as a systematic reminding measure. The 14 patients who were disturbed by the reminder gave the following reasons: phone call too early in the morning (n = 1), were waiting for important results (n = 1), were reached at work (n = 4), had the feeling of being treated as senile (n = 2), felt it was not necessary (n = 3), other reasons (n = 3).

### Economic evaluation of the intervention

The intervention generated 55 additional consultations: 40 re-scheduled consultations took place in the intervention group, and 15 consultations were booked for other patients after cancellation. The net financial benefit of the intervention was estimated to be 1850. - EUR over 3 months (provide 2010 inflation-corrected values), once costs linked to the intervention were deducted (Table 4).

**Table 4:** Economic evaluation of the intervention

Intervention group	Costs (EUR)	Benefits (EUR)
55 additional consultations *(average consultation 30 min; average charge 80 EUR* *- 40 consultations saved* *- 15 new consultations allocated on opened scheduling slots due to cancellation*		4412.-

Telecommunication cost		
Phone (8 cts/call)	61.-	
SMS (8 cts/txt)	8.-	

Letter (84 cts.-/mail)	157.-	

Research assistant salary for 3 months	2340.-	

Total cost	2566.-	
Total benefits		4412.-

Net benefits	1846.-	

## Discussion

This intervention design, sequentially using phone calls, SMS and postal reminder, proved to be efficient, cost-effective, and well accepted by patients, in an urban primary care clinic serving a majority of vulnerable population.

The intervention significantly reduced the rate of missed appointments. However, subgroup analysis shows that the intervention's effectiveness was only statistically significant in the primary care and the tobacco cessation consultations. While reasons of the lack of efficacy of the intervention in the HIV and dietician consultations are not clear, one explanation could be attributable to low numbers of patients enrolled in the HIV (n = 303) and in the dietician consultation (n = 127). Other characteristics such as patient profiles or patterns in profiles may also have impacted on such differences. Analysis of sociodemographic and medical characteristics of patients attending either the general/nicotine cessation consultation versus the HIV consultation showed that there was a significantly higher percentage of male, insured patients, follow-up consultations of < 1 year, consultations made by senior doctors in the HIV consultation.

The overall 11.4% rate of missed appointments in the control group was lower than estimated based on prior assessments. This fact could be explained in several ways: the rate of missed appointments may have been previously overestimated on the basis of administrative indicators which did not distinguish between missed and cancelled appointments. Seasonal variations in attendance rate have also been described [18] and an unpublished report about missed appointments in our clinic showed that, in 2001, rates of missed appointments varied between 23.5%, 35% and 16% at three different periods of the year (June, August and March) [19]. However, most probably, the implementation of the intervention itself may have had a positive influence on both intervention and control patients, through information panels and may have increased alertness and awareness of receptionists and patients. A year after the end of the study, the average rate of missed appointments was back to 14%, which calls for the inclusion of such strategies in the administrative routine of outpatient clinics.

Patients enrolled in the HIV outpatient care and in the tobacco cession program had higher baseline rates of missed appointments. Previous studies showed that overall attendance rates appear to be rather low in such populations: up to 30% of HIV infected patients scheduled for a clinic appointment never turned up for an initial evaluation [20,21] and 35% of scheduled medical appointments were not honoured in an other setting [22]. Optimal attendance can reach 66 to 83% of patients enrolled in nicotine cessation program [23-25].

The success of such an intervention depends on the mobile phone and phone penetration and recording in a given population [26,27]. In our population, only 51% of the patients had their mobile phone recorded at the clinic and 6% both a mobile and a fixed phone. It is not possible to estimate on such basis penetration rates of mobile phones among our patient population since patients may prefer to give only one phone number. However, while penetration rates of mobile phone range from 20% to 99% over the world (calculated as the% of total telephone subscribers), in Switzerland, mobile phone subscribers represented 64.6% of total telephone subscribers in 2008 [27]. Recent studies showed that, whereas phone calls and SMS are equally effective in reducing the rate of missed appointments in various settings, SMS are more cost-effective [15,16]. SMS requires significantly less staff resources: messages can be standardised and sent to a large number of patients at low cost [14]. In our setting, the fact that about half of the patients did not provide us with their mobile phone number may have made an intervention exclusively designed on the basis of a SMS reminder less effective than the sequential intervention used. Thus, careful analysis of mobile and fixed phone distribution among patients is essential before choosing and implementing a reminder system.

Missed appointments were significantly associated with younger age, being an asylum seeker, having substance abuse problems, having a follow-up appointment after more than a year These findings are in accordance with prior studies: factors influencing missed appointments usually include patient related factors such young age [7,28,29], poor socioeconomic status, and health insurance coverage [5,10,30,31]. Structural aspects also play a role: a long interval between the booking time and the time of the consultation, frequent changes inside the medical team with lack of interpersonal continuity, and difficulty to contact the clinic to cancel or report the appointment, have been identified as factors predicting missed appointments [4].

Conclusions are conflicting concerning gender, some studies showing a higher rate of missed appointments in men [4] while others in women [28]. Although most authors observe that patients from lower socioeconomic level or from deprived areas tend to have an increased rate of missed appointments [4,7,26,28], the fact that being an undocumented immigrant was not associated with missed appointments in our study indicates that low socioeconomic status may hide other difficulties such as linguistic, communication or psychosocial issues, and may not be in itself a predictor of missed appointments. Interestingly and contrary to previous studies, being a new patient or having a post-ED visit was not associated with missed appointments [7,32]. Junior doctors' patients were more likely to miss their appointments than senior doctors' patients. These findings support the assumption that interpersonal continuity may increase attendance rate and build up loyalty, trust and respect [7,33]. Junior doctors tend to stay for one year of training at our primary care clinic, while senior doctors represent a more stable staff.

Our study has several limitations. First, we did not compare different reminder systems and, therefore, are not able to identify which one works better than the other and for which population of patients. Instead, we chose to test a pragmatic sequential intervention, taking into account the difficulties in reaching our patient population. Second, we are missing information about 57 (8.7%) patients scheduled for an appointment in the intervention group and 93 (5.4%) patients scheduled for an appointment in the control group. These patients were included in the analysis based on intention to treat. Reallocation of free slots was not recorded for appointments cancelled more than 48 hours before the appointments in both intervention and control groups. Finally, the acceptability survey was made by phone and reached only patients who had a phone and responded at the first attempt. It did not distinguish between patients reached by phone, SMS or mail, but one could expect that people at work would be more disturbed by a phone call than by a SMS. Finally, the economic evaluation, which, proved to be economically efficient, similarly to other randomised studies testing text messaging and/or mobile phone reminders only, [2,11,16,34] was limited. It did not take into account the precise number of missed appointments which led to subsequent rebooking. Economic implications may differ according to the health care system: wasted physician time may be more relevant in countries based on a capitation payment system while financial losses may raise more concerns in health care systems based on a fee-for-service model.

## Conclusions

A strategy of sequential interventions successively using phone calls, SMS and postal reminder, proved to be effective and economically efficient in a population where mobile phone penetration was only 61.7%; it also was generally well accepted by the patients who found it useful. Nevertheless, it will require further evaluation once generalised and integrated into routine practice.

## Competing interests

The authors declare that they have no competing interests.

## Authors' contributions

NJP and MDD conceived and designed the study, coordinated the data collection and NJP wrote the first draft of the manuscript. CC collected the data, synthesized the results and helped to draft the manuscript. MPK participated to coordination of the study, and carried out the data analysis. VM participated to study conception, design and coordination of the study. AC participated to the coordination of the study in the HIV clinic and helped to draft the manuscript. JMG helped to the design and funding of the study and critically revised manuscript. All authors read and approved the final manuscript.

## Pre-publication history

The pre-publication history for this paper can be accessed here:

http://www.biomedcentral.com/1471-2296/11/79/prepub
